# Factors related to white blood cell elevation in acute type A aortic dissection

**DOI:** 10.1371/journal.pone.0228954

**Published:** 2020-02-06

**Authors:** Keito Suzuki, Naoyuki Kimura, Makiko Mieno, Daijiro Hori, Akira Sezai, Atsushi Yamaguchi, Masashi Tanaka

**Affiliations:** 1 Department of Cardiovascular Surgery, Nihon University, Itabashi-ku, Tokyo, Japan; 2 Department of Cardiovascular Surgery, Saitama Medical Center, Jichi Medical University, Omiya-ku, Saitama, Japan; 3 Department of Medical Informatics, Center for Information, Jichi Medical University, Shimotsuke, Japan; IRCCS Policlinico S.Donato, ITALY

## Abstract

Aortic dissection may induce a systemic inflammatory reaction. The etiological backgrounds for elevation of the white blood cell count remain to be clarified. In 466 patients with acute type A aortic dissection treated surgically within 48 hours of symptom onset, the etiologic background of an elevated admission white blood cell count and the effect of such elevation on outcomes were assessed retrospectively. Patients’ white blood cell count differed significantly in relation to the extent of dissection, with a median (25th, 75th percentile) white blood cell count of 10.4 (8.1, 13.9) x 10^3^/μL for dissection confined to the ascending aorta, 10.5 (8.2,13.) 10^3^/μL for dissection extending to the aortic arch/descending aorta, 11.1 (8.2, 13.7) x 10^3^/μL for extension to the abdominal aorta, and 13.3 (9.8, 15.9) x 10^3^/μL for extension to the iliac artery (p<0.001). With 11.0 x 10^3/^μL used as the cut-off value for white blood cell count elevation, multivariable analysis showed current smoking (p<0.001; odds ratio, 2.79), dissection extending to the iliac artery (p = 0.006; odds ratio, 1.79), age (p = 0.007, odds ratio, 0.98), and no coronary ischemia (p = 0.027, odds ratio, 2.22) to be factors related to the elevated white blood cell count. Mean age differed significantly between patients with and without an elevated white blood cell count (62.3 vs. 68.3 years, p <0.001). Although in-hospital mortality was similar (7.5% vs.10.9%, p = 0.19), 5-year survival was lower in patients without an elevated count (85.7% vs. 78.6%, p = 0.019), reflecting their more advanced age. In conclusion, our data suggest that dissection morphology and patient age influence the acute phase systemic inflammatory response associated with an elevated white blood cell count in patients with ATAAD. A better understanding of this relation may help optimize diagnosis and perioperative care.

## Introduction

Acute aortic dissection is a life-threatening cardiovascular emergency that occurs when an intimal tear leads to sudden influx of blood flow between layers of the aortic wall, creating a false lumen (FL) that propagates longitudinally in both the proximal and distal directions. In patients with acute type A aortic dissection (ATAAD), coexisting organ ischemia and hemodynamic instability strongly influence outcomes [1–4]; thus, appropriate management is critically important. Acute aortic dissection may induce systemic inflammatory reactions. Regarding mechanism of pathogenesis, chronic inflammation of the medial layer of the aortic wall has been reported to cause aneurysm growth, leading to aortic dissection [5–7]. In addition, blood flow into the FL within the aortic wall may induce recruitment of immune cells. In an animal model, inflammation of the adventitia characterized by neutrophil recruitment was shown to promote tissue damage, leading to aortic dilation and rupture [8]. Moreover, a recent study of surgically resected aortic specimens revealed association between activation of STAT 3 in adventitial tissue and neutrophil infiltration [9].

Clinical studies involving patients with acute type B aortic dissection have shown C-reactive protein elevation to be a predictor of early impairment of oxygenation [10] and of poor late survival [11]. In patients with ATAAD, hemodynamic instability can occur as a result of cardiac tamponade or aortic insufficiency, and emergency aortic repair under cardiopulmonary bypass induces a strong perioperative inflammatory response, making investigation of the early inflammatory response more complicated. Nevertheless, there is evidence that systemic inflammation characterized by elevation of the white blood cell (WBC) count [12–14] or an increased neutrophil/lymphocyte ratio [13, 15, 16] influences outcomes in patients with ATAAD. Although biomarkers of disordered coagulation and fibrinolysis D-dimer and fibrin degradation products have been well studied in relation to acute aortic dissection [17–19], WBC count elevation and its etiology in cases of acute aortic dissection have not been fully elucidated, and the relation between WBC elevation and dissection morphology is poorly understood.

We performed a retrospective study of patients with ATAAD who underwent aortic repair within 48 hours of symptom onset to investigate the acute phase inflammatory response in terms of the WBC count, etiologic background of WBC count elevation, and the effect of such elevation on outcomes.

## Materials and methods

The study included 466 patients with ATAAD who underwent emergency aortic repair at Saitama Medical Center, Jichi Medical University (Saitama, Japan) between January 2008 and April 2018 or at Nihon University Hospital (Tokyo, Japan) between January 2014 and January 2018. The patients were identified through a search of the aortic databases of the 2 hospitals, which together contained the records of 500 patients who had undergone such emergency repair. Thirty-four of the patients had undergone the repair more than 48 hours after symptom onset because they did not seek medical attention immediately or because referral from the local hospital was not immediate, and these patients were thus not included in the study. The aortic dissection was classified as acute if symptom onset was within 2 weeks of the patient’s presentation. ATAAD was diagnosed by computed tomography (CT) in all patients. The institutional review board at each of the centers approved the study (approval number: Saitama Medical Center, Jichi Medical University; S19-043, Nihon University; RK-190906-1) and waived the need for informed consent.

**Fig 1:**
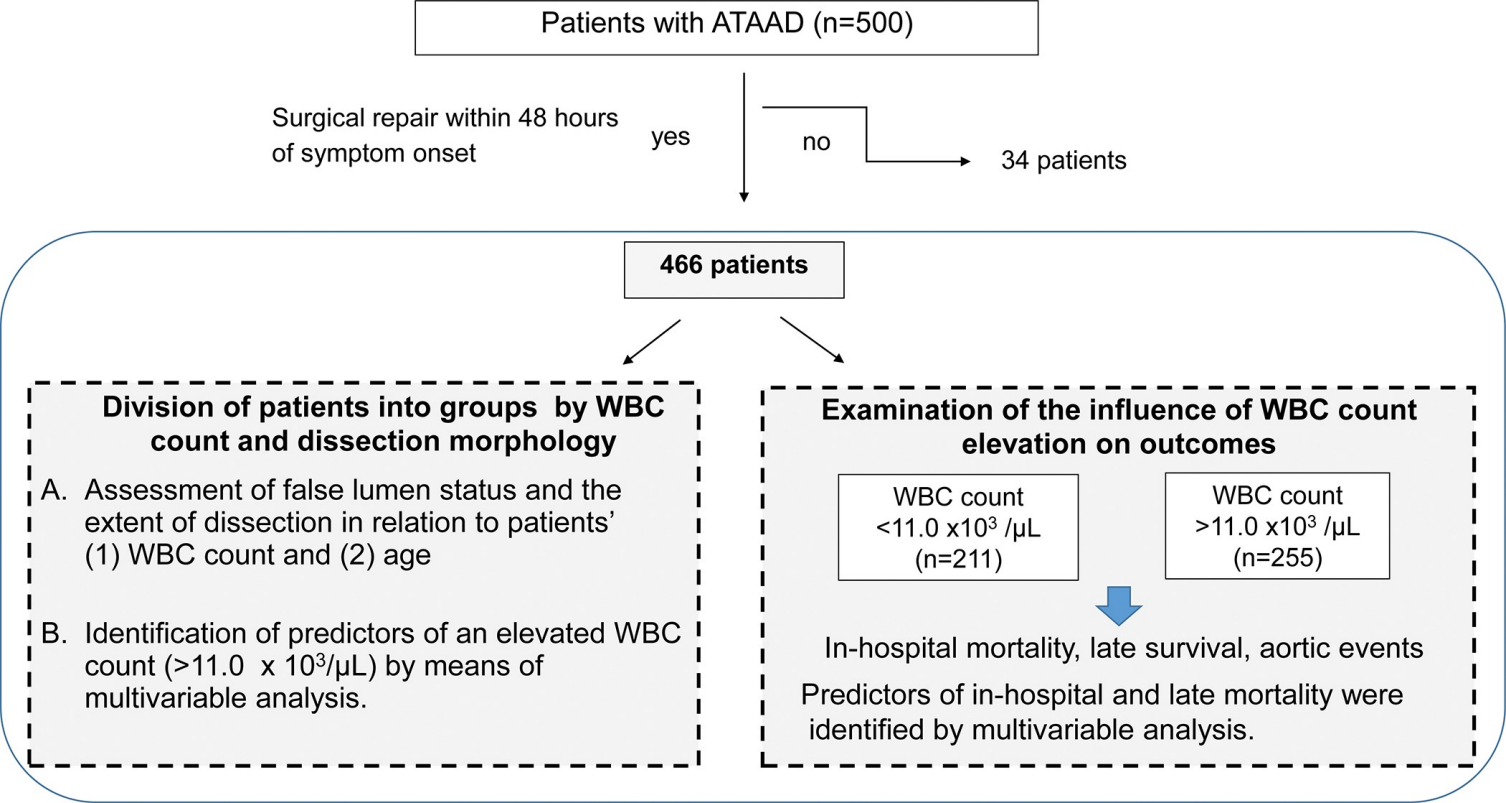
Flow diagram of the study, including patient selection and factors addressed. ATAAD, acute type A aortic dissection; WBC, white blood cell.

A flow diagram of the study, including patient selection and items examined is given in Fig 1. Patients’ hospital records were searched for clinical information, especially WBC and imaging findings on admission. On the basis of results of the largest reported study of WBC counts in patients with aortic dissection [14], 11.0 x 10^3^/μL was selected as the cut-off value for discriminating between normal and elevated WBC counts. Patients were divided into 2 groups: those with a WBC count ≤11.0 x 10^3^/μL (normal WBC count group) and those with a WBC count >11.0 x 10^3^/μL (elevated WBC count group). We first investigated etiologic factors for WBC elevation. Two morphologic variables, FL status and the distal extent of dissection, were assessed in relation to patients’ WBC count and age on admission. We then investigated the influence of WBC count elevation on outcomes. Early outcomes (in-hospital mortality and morbidity) and late outcomes (survival and aortic event-free survival) were compared between the 2 patient groups.

FL status and the distal extent of dissection were classified as reported previously [18]. Briefly, preoperative contrast-enhanced CT imaging data were independently reviewed by 2 of the authors (KS and NK), who are cardiovascular surgeons. FL status was judged from delayed-phase CT findings or from early-phase CT findings when no delayed-phase CT scans had been obtained. The FL was classified as thrombosed (n = 112), partially thrombosed (n = 102), or patent (no thrombosis) (n = 252). A thrombosed FL with a small intimal tear (ulcer-like projection) was classified as “thrombosed.” Patients were divided into the following 4 groups according to the extent of dissection: dissection limited to the ascending aorta (DeBakey type II dissection, n = 42), dissection extending to the aortic arch or descending thoracic aorta (n = 91), dissection extending to the abdominal aorta (n = 142), and dissection extending to 1 or both iliac arteries (n = 191).

The operative techniques used for emergency aortic repair were as reported previously and similar, whether the repair was performed at Saitama Medical Center or Nihon University Hospital [2, 18, 20]. Briefly, the tear-oriented approach was applied, i.e., replacement of the ascending aorta or hemiarch replacement combined with resection of the primary intimal tear was performed with an open distal anastomosis under moderate to deep hypothermia (rectal temperature: 20–25°C). If the intimal tear was located in the aortic arch or proximal descending aorta, partial arch replacement (reconstruction of 1 or 2 supra-arch branches) or total arch replacement was performed under selective anterograde cerebral perfusion. The frozen elephant trunk procedure was applied for total arch replacement in patients with a narrowed true lumen in the downstream aorta. The proximal stump was trimmed and reinforced with Teflon felt. Glue (fibrin glue or BioGlue) was used between dissected layers in some patients, according to the surgeon’s preference. Modified non-valve-sparing Bentall aortic root replacement or valve-sparing re-implantation was performed in patients with aortic root dilation or an intimal tear located at the aortic root.

### Statistical analysis

Results are presented as the number (percentage) of patients, as mean±standard deviation values, or as median (25th, 75th percentile) values. FL status and the distal extent of dissection were examination in relation to age and to WBC cell count, and differences between groups were analyzed by Kruskal-Wallis test. Multivariable forward stepwise logistic regression analysis was performed to identify factors related to WBC count elevation (>11.0 x 10^3^/μL) and in-hospital mortality. The following variables were entered into the analysis: age, male sex, obesity (body mass index >30 kg/m^2^), bicuspid aortic valve, Marfan syndrome, current smoking, chronic obstructive pulmonary disease, hypertension, diabetes, history of ischemic heart disease, history of cerebrovascular disease, hemodialysis, history of cardiac surgery, shock (systolic blood pressure <80 mmHg), severe aortic insufficiency, organ ischemia (brain, coronary, mesenteric, or lower extremity), distal extent of dissection (ascending aorta, descending thoracic aorta, abdominal aorta, or iliac artery), FL status (thrombosed, partially thrombosed, or patent), and WBC count >11.0 x 10^3^/μL (for in-hospital mortality). Differences in variables between patients in the normal WBC count group and those in the elevated WBC count group were analyzed by chi-square or Fisher’s exact test or by unpaired *t*-test or Mann-Whitney *U* test, as appropriate. An aortic event was defined as redissection, reoperation, aortic rupture, or sudden death. Freedom from death or aortic events was estimated by the Kaplan-Meier method, and differences between the groups were analyzed by log-rank test. The mean follow-up period was 41.9±31.4 months. Forward stepwise Cox proportional hazards regression analysis was performed to identify factors related to late mortality among hospital survivors. A WBC count >11.0 x 10^3^/μL was included as a factor in this analysis. All statistical analyses were performed with SPSS 26.0 for Windows software (IBM Corp, Armonk, NY, USA), and p<0.05 was considered significant.

## Results

### WBC count and dissection morphology

**Fig 2:**
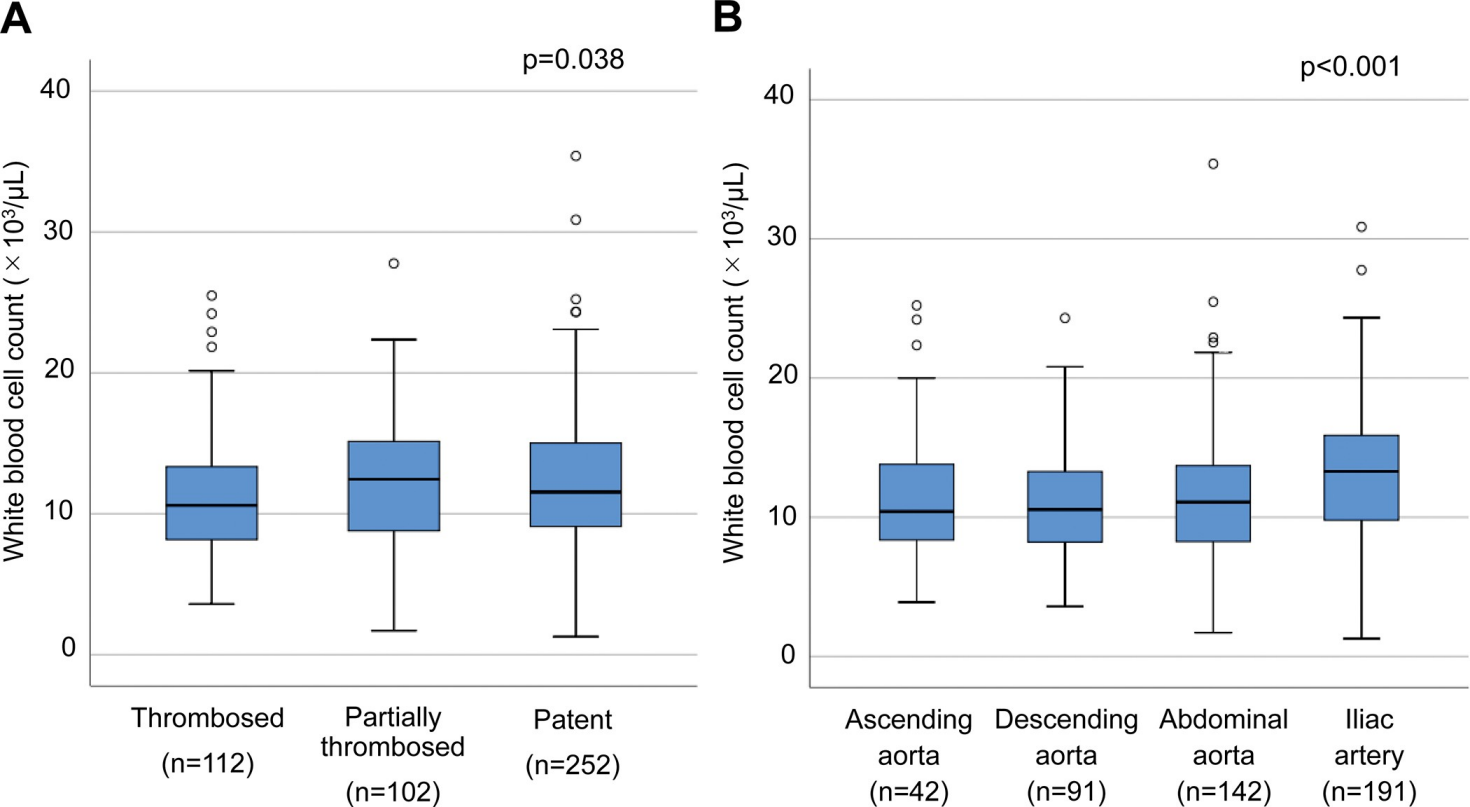
Box and whisker plots of morphologic classifications in relation to white blood cell count. (A) False lumen status. (B) Extent of aortic dissection. Probability values were obtained by Kruskal-Wallis test.

**Fig 3:**
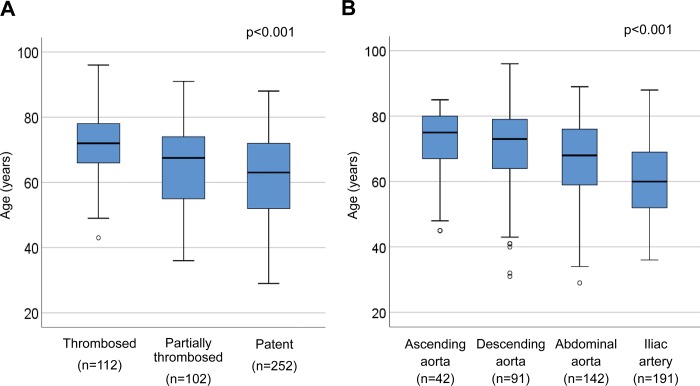
Box and whisker plots of morphologic classifications in relation to age. (A) False lumen status. (B) Extent of aortic dissection. Probability values were obtained by Kruskal-Wallis test.

The mean and median admission WBC counts were 11.4±4.6 x 10^3/^μL and 11.4 (8.7, 14.9) x 10^3/^μL, respectively, with 54.7% of the patients (255/466) having a WBC count >11.0 x 10^3^/μL. In investigating the relation between dissection morphology and WBC count, we found the median count to be 10.6 (8.1, 13.4) x 10^3^/μL in the thrombosed FL group, 12.4 (8.8, 15.2) x 10^3^/μL in the partially thrombosed FL group, and 11.5 (9.1, 15.0) x 10^3^/μL in the patent FL group (Fig 2A, p = 0.038). When patients were classified by the distal extent of dissection, the median WBC count was 10.4 (8.1, 13.9) x 10^3^/μL for patients in whom the dissection extended to the ascending aorta, 10.5 (8.2, 13.3) μg/mL for patients in whom the dissection extended to the aortic arch or descending thoracic aorta, 11.1 (8.2, 13.7) x 10^3^/μL for patients in whom the dissection extended to the abdominal aorta, and 13.3 (9.8, 15.9) x 10^3^/μL for patients in whom the dissection extended to the iliac artery (Fig 2B, p<0.001). In assessing the relation between dissection morphology and age, we found, as shown in Fig 3, that FL status and the distal extent of dissection differed significantly in relation to age. Elderly patients were more likely to have a thrombosed FL lumen and dissection limited to the thoracic aorta.

### Characteristics of patients with an elevated WBC count

Clinical presenting and dissection characteristics of the patients with a WBC count ≤11.0 x 10^3^/μL (normal WBC count group) and those with a WBC count >11.0 x 10^3^/μL (elevated WBC count group) are shown in Table 1. Age differed significantly between these 2 groups (68.3 years in the normal WBC count group and 63.3 years in the elevated WBC count group, p<0.001). Patients with an elevated WBC count were more likely than those in the normal WBC count group to be male and smokers. As presenting characteristics, abdominal pain and mesenteric ischemia were significantly more prevalent in the elevated WBC count group than in the normal WBC count group. However, the prevalence of preoperative shock and that of syncope were similar between the 2 groups. A thrombosed FL was significantly less prevalent in the elevated WBC count group, and dissection extending to the iliac artery was significantly more prevalent in this group. Admission laboratory data for the 2 groups are shown in Table 2. The hemoglobin concentration, hematocrit, and platelet count were significantly higher in the elevated WBC count group than in the normal WBC count group. Moreover, lactase dehydrogenase, total bilirubin, and D-dimer concentrations were significantly increased in this group, as was the activated partial thromboplastin time.

**Table 1:** Patients’ clinical characteristics, presenting characteristics, and dissection characteristics, per normal and elevated white blood cell counts (≤11.0 x10^3^/μL vs. >11.0 x10^3^/μL).

	WBC count ≤11.0 x10^3^/μL (n = 211)	WBC count >11.0 x10^3^/μL (n = 255)	p Value
**Clinical characteristics**			
Age (years)	68.3 ± 12.6	62.3 ± 12.5	<0.001
Sex, male	97 (46.0%)	154 (60.4%)	0.002
Obesity (BMI >30)	12 (5.7%)	25 (9.8%)	0.10
Bicuspid aortic valve	4 (1.9%)	5 (2.0%)	1.0
Marfan syndrome	4 (1.9%)	3 (1.6%)	1.0
Current smoking	38 (18.0%)	106 (41.6%)	<0.001
COPD	6 (2.8%)	8 (3.1%)	0.85
Hypertension	152 (72.0%)	195 (76.5%)	0.28
Diabetes	16 (7.6%)	22 (8.6%)	0.68
Dyslipidemia	33 (15.6%)	53 (20.8%)	0.15
History of IHD	8 (3.8%)	12 (4.7%)	0.63
History of CVD	19 (9.0%)	22 (8.6%)	0.89
Hemodialysis	8 (3.8%)	2 (0.8%)	0.056
History of cardiac surgery	4 (0.9%)	0 (0%)	0.088
**Presenting characteristics**			
Chest pain and/or back pain	176 (83.4%)	213 (83.5%)	0.97
Abdominal pain	7 (3.3%)	21 (8.2%)	0.026
Syncope	47 (22.3%)	46 (18.0%)	0.26
Shock (systolic BP <80 mmHg)	51 (24.2%)	66 (25.9%)	0.67
Severe aortic insufficiency	17 (8.1%)	14 (5.5%)	0.27
Organ ischemia			
Brain	33 (15.6%)	27 (10.6%)	0.11
Heart	23 (10.9%)	16 (6.3%)	0.073
Mesentery	6 (2.8%)	19 (7.5%)	0.028
Lower extremity	23 (10.9%)	41 (16.1%)	0.11
**Dissection characteristics**			
False lumen status			
Thrombosed	63 (29.9%)	49 (19.2%)	0.007
Partially thrombosed	38 (18.0%)	64 (25.1%)	0.065
Patent	110 (52.1%)	142 (55.7%)	0.44
Extent of dissection			
Ascending aorta	24 (11.4%)	18 (7.1%)	0.11
Aortic arch or descending aorta	53 (25.1%)	38 (14.9%)	0.006
Abdominal aorta	69 (32.7%)	73 (28.6%)	0.34
Iliac artery	65 (30.8%)	126 (49.4%)	<0.001
Primary entry site^a^			
Ascending aorta	133 (63.0%)	133 (52.2%)	0.018
Aortic arch	40 (19.0%)	49 (19.2%)	0.94
Descending aorta or unknown	42 (19.9%)	75 (29.4%)	0.018

Data are shown as mean ± standard deviation or number (percentage) of patients.

^a^Multiple entry sites existed in some patients.

WBC, white blood cell; BMI, body mass index; COPD, chronic obstructive pulmonary disease; IHD, ischemic heart disease; CVD, cerebrovascular disease; BP, blood pressure.

**Table 2:** Patients’ admission laboratory values, per white blood cell count (≤11.0 x10^3^/μL vs. >11.0 x10^3^/μL).

	WBC count ≤11.0 x10^3^/μL (n = 211)	WBC count >11.0 x10^3^/μL (n = 255)	p Value
WBC count (x 10^3^/μL)	8.5 (6.9, 9.8)	14.4 (12.8, 16.9)	<0.001
Hemoglobin (g/dL)	12.2 (10.8, 13.3)	13.0 (11.8, 14.2)	<0.001
Hematocrit (%)	36.1 (32.2, 39.5)	38.3 (35.1, 42.2)	<0.001
Platelet (x 10^4^/μL)	17.0 (14.0, 20.7)	19.1 (15.7, 23.0)	<0.001
Albumin (g/dL)	3.7 (3.4, 4.1)	3.8 (3.5, 4.1)	0.16
Total bilirubin (mg/dL)	0.61 (0.44, 0.82)	0.71 (0.51, 0.97)	0.001
LDH (IU/L)	245 (205, 301)	262 (227, 322)	0.003
AST (IU/L)	26 (20, 40)	26 (20, 43)	0.94
ALT (IU/L)	19 (13, 42)	22 (14, 41)	0.063
Creatinine (mg/dL)	0.84 (0.66, 1.09)	0.9 (0.7, 1.14)	0.38
eGFR (mL/min/1.73m^2^)	59.5 (47.0, 75.1)	62.3 (46.9, 78.8)	0.25
PT-INR	1.05 (0.98, 1.14)	1.07 (1.0, 1.14)	0.50
APTT (sec)	32.9 (29.5, 36.7)	34.0 (29.9, 39.7)	0.037
D-dimer^a^ (mg/mL)	18.0 (6.7, 58.5)	30.1 (9.8, 94.4)	0.005

Data are shown as median (25th, 75th percentile) values.

^a^Data were obtained for 91% (424/466) of the patients.

WBC, white blood cell; LDH, lactase dehydrogenase; AST, aspartate aminotransferase; ALT, alanine aminotransferase; eGFR, estimated glomerular filtration rate; PT-INR, international normalized ratio of prothrombin time; APTT, activated partial thromboplastin time.

### Factors related to WBC count elevation

Multivariable analysis showed current smoking, dissection extending to the iliac artery, age, and absence of coronary ischemia to be factors related to an elevated WBC count (Table 3).

**Table 3:** Factors related to WBC count elevation (>11,000/μL), as determined by multivariable analysis.

	p Value	Odds ratio	95% CI
Current smoking	<0.001	2.79	1.77–4.39
Dissection extending to the iliac artery	0.006	1.79	3.60–26.73
Age	0.007	0.98	0.96–0.99
Absence of coronary ischemia	0.027	2.22	1.09–4.54

CI, confidence interval.

### Influence of WBC count elevation on outcomes

**Fig 4:**
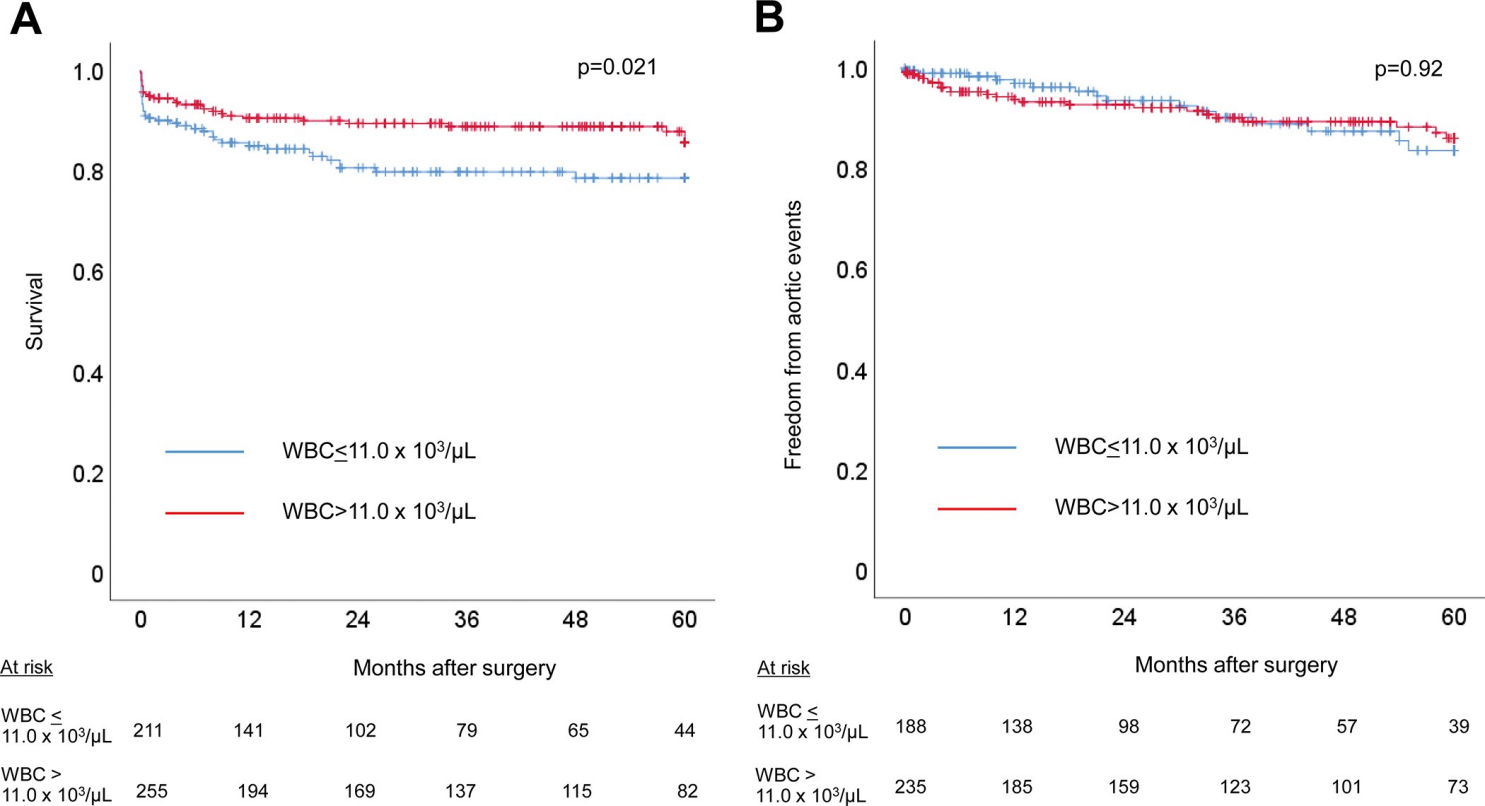
Kaplan-Meier curves of (A) cumulative survival of ATAAD patients, per WBC count (≤11.0 x 10^3^/μL or >11.0 x 10^3^/μL) and of (B) freedom from aortic events after discharge, per WBC count (≤11.0 x 10^3^/μL or >11.0 x 10^3^/μL). Probability values were obtained by log-rank test. ATAAD, acute type A aortic dissection; WBC, white blood cell.

To assess the influence of an elevated WBC count on outcomes, we compared operative variables and outcomes between the groups. Operative variables and early outcomes are shown for the normal WBC count group and elevated WBC count group in Table 4. Operation time was significantly longer in the elevated WBC count group than in the normal WBC group, but the procedures performed and other operative variables did not differ between the 2 groups. The overall 30-day mortality and in-hospital mortality were 7.5% (35/466) and 9.0% (42/466), respectively. Thirty-day mortality was 10.0% (21/211) in the normal WBC count group and 5.5% (14/255) in the elevated WBC count group (p = 0.069). In-hospital mortality was 10.9% (23/211) in the normal WBC count group and 7.5% (19/255) in the elevated WBC count group (p = 0.19). Similarly, the incidence of postoperative complications did not differ between the 2 groups. Details of in-hospital mortality are shown in Table 5. There were no differences in the causes of in-hospital mortality between the 2 groups. Multivariable logistic regression analysis performed for in-hospital mortality showed obesity (p<0.001, odds ratio: 6.92, 95% confidence interval: 2.56–18.69), shock (p<0.001, 6.31, 2.89–13.76), mesenteric ischemia (p<0.001, 10.5, 3.64–30.61), brain ischemia (p = 0.01, 2.97, 1.29–6.85), and coronary ischemia (p = 0.015, 3.17, 1.26–8.02) to be factors related to in-hospital mortality. Late outcomes are shown in Fig 4. Five-year survival was 78.6±3.2% in the normal WBC count group versus 85.7±2.7% in the elevated WBC count group (p = 0.021), whereas 5-year freedom from aortic events was 83.6±4.1% in the normal WBC count group and 86.0±2.9% in the elevated WBC count group (p = 0.92). The details of late mortality are listed in Table 5. As with in-hospital mortality, there were no differences in the causes of late mortality between the 2 groups. Cox proportional hazards regression analysis identified age (p<0.001, hazard ratio: 1.102, 95% confidence interval: 1.052–1.152), a history of ischemic heart disease (p = 0.007, 4.53, 1.52–13.49), and Marfan syndrome (p = 0.015, 15.1, 1.7–132.4) as factors related to late mortality.

**Table 4:** Operative variables and early outcomes, per white blood cell count (≤11.0 x10^3^/μL vs. >11.0 x10^3^/μL).

	WBC count ≤11.0 x10^3^/μL (n = 211)	WBC count >11.0 x10^3^/μL (n = 255)	p Value
**Operative variables**			
Proximal reconstruction			
Valve re-suspension	188 (89.1%)	235 (92.2%)	0.26
Modified Bentall procedure	13 (6.2%)	7 (2.6%)	0.070
Valve-conserving root surgery	1 (0.5%)	2 (0.8%)	1.0
Isolated aortic valve replacement	9 (4.3%)	11 (4.3%)	0.98
Distal extent of aortic resection			
Ascending aorta/hemiarch	169 (80.1%)	193 (75.7%)	0.26
Aortic arch	42 (19.9%)	62 (24.3%)	0.26
Open stent insertion	5 (2.4%)	13 (5.1%)	0.13
Coronary artery bypass grafting	14 (6.6%)	20 (7.8%)	0.62
Resection of primary entry site	156 (73.9%)	180 (70.2%)	0.47
Operation time (minutes)	310 (249, 391)	329 (270, 431)	0.024
CPB time (minutes)	135 (111, 182)	138 (116, 201)	0.14
Myocardial ischemia time (minutes)	91 (75, 119)	95 (78, 129)	0.10
Blood loss (mL)	905 (565, 1527)	860 (560, 1400)	0.98
Lowest body temperature (°C)	20.0 (19.4, 23.7)	20.3 (19.7, 23.9)	0.87
**Early outcomes**			
Death within 30 days of surgery	21 (10.0%)	14 (5.5%)	0.069
In-hospital death	23 (10.9%)	19 (7.5%)	0.19
Length of hospital stay (days)	7.2±8.0	8.3±7.3	0.14
**Complications**			
New-onset postoperative stroke	15 (7.2%)	26 (10.2%)	0.25
Prolonged ventilation (>48 hours)	102 (48.3%)	133 (52.2%)	0.41
Re-exploration for bleeding	12 (5.7%)	7 (2.7%)	0.11
Mediastinitis	2 (0.9%)	5 (2.0%)	0.61
Renal replacement therapy	17 (8.1%)	30 (11.8%)	0.19

Data are shown as mean±standard deviation, median (interquartile range) values, or number (percentage) of patients.

WBC, white blood cell; CPB, cardiopulmonary bypass.

**Table 5:** Mortality and its causes in the total patients and per white blood cell count (<11.0 x10^3^/μL vs. >11.0 x10^3^/μL).

	Total patients	WBC count ≤11.0 x10^3^/μL	WBC count >11.0 x10^3^/μL	p Value
**In-hospital mortality**	n = 42	n = 23	n = 19	
Multi-organ failure	20 (47.6%)	8 (34.8%)	12 (63.2%)	0.067
CVD	10 (23.8%)	6 (26.1%)	4 (21.1%)	0.98
Heart failure	9 (21.4%)	6 (26.1%)	3 (15.8%)	0.67
Bleeding	3 (7.1%)	3 (13.0%)	0 (0%)	0.30
**Late mortality**	n = 31	n = 17	n = 14	
Pneumonia	11 (35.5%)	5 (29.4%)	6 (42.9%)	0.43
Heart failure	5 (16.1%)	5 (29.4%)	0 (0%)	0.085
Aortic rupture/sudden death	5 (16.1%)	1 (5.9%)	4 (28.6%)	0.22
Multi-organ failure	4 (12.9%)	3 (17.6%)	1 (7.1%)	0.74
CVD	3 (9.7%)	1 (5.9%)	2 (14.3%)	0.43
Malignancy	2 (17.4%)	1 (5.9%)	1 (7.1%)	1.0
Renal failure	1 (3.2%)	1 (5.9%)	0 (0%)	1.0

Data are shown as number (percentage) of patients.

WBC, white blood cell; CVD, cerebrovascular disease.

## Discussion

Elevation of the WBC count is a well-known acute phase reaction in patients with aortic dissection and several studies have already investigated the effect of WBC count elevation in ATAAD [12–14]. Fan et al. used a cut-off value of 11.0 x 10^3^/μL to define WBC elevation and reported that in-hospital mortality was worse for patients with an elevated WBC count (n = 216) than for patients with a normal WBC count (n = 354) [14]. Although the Fan et al. study was the largest investigation of the inflammatory response in patients with ATAAD (n = 570), the study included patients admitted more than 48 hours after symptom onset [14]. In the study described herein, we focused on the acute phase inflammatory response by excluding patients hospitalized more than 48 hours after symptom onset. Our study showed that dissection morphology and age have some influence the WBC count in patients with ATAAD.

The mechanism of the acute inflammatory response in aortic dissection has been studied in animal models. Kurihara et al. established an *in vivo* model of aortic dissection by administration of angiotensin II to mice treated with a lysyl oxidase inhibitor [21], and they reported that matrix metalloproteinase-9 released by infiltrating neutrophils may be involved in the development of dissection [21]. In the same animal model, Anzai et al. reported that acute aortic dissection triggers expression of CXCL1 and G-CSF in the adventitia of the dissecting aorta, leading to elevation of circulating CXCL1/G-CSF levels [8]. These chemokines promote mobilization of neutrophils from the bone marrow to the peripheral blood [8]. In addition to CXCL1 and G-CSF, multiple other cytokines and chemokines, including IL-6 [22, 23] and MCP-1 [23], have been reported to play a role in the local and systemic inflammatory responses after acute aortic dissection. These findings suggest that anti-inflammatory therapy may be useful for preventing aortic dissection in high-risk patients, including those with aortic dilatation or bicuspid aortopathy. Our study also showed association between the distal aortic dissection and the WBC count, possibly reflecting increased production of chemoattractants by the affected aorta in patients with more extensive dissection. Interestingly, patients with a thrombosed FL had relatively low WBC count. We assume that FL thrombosis decreases blood flow from the false to true lumen and attenuates efflux of chemoattractants from the affected aorta. Our study patients with a partially thrombosed FL had the highest median WBC count. It has been reported that thrombogenicity and fibrinolytic activity of a partially thrombosed FL are increased, in comparison to those of a patent or thrombosed FL [18, 24]. Observational studies of deep vein thrombosis have shown that inflammatory cells accumulate within the thrombus and surrounding vein wall during the natural history of the disease [25]. In a mouse model of deep vein thrombosis, toll-like receptor 9 was shown to play a role in thrombus resolution [26]. We think that increased thrombogenicity in patients with a partially thrombosed FL might have led to the WBC count elevation in this group.

Chronic elevation of inflammatory markers has been found in various age-related diseases, including atherosclerosis and Alzheimer’s disease [27, 28], and aging-associated inflammation may lead to dysregulation of innate immunity [29]. However, the effect of aging on the acute inflammatory response to critical illness is not fully understood. Kale et al. studied patients hospitalized with community acquired pneumonia, divided them into groups by age (<50, 51–64, 65–74, 75–84, and ≥85 years), and reported no age-related differences in inflammatory markers, including TNF, IL-6, and IL-10 [30]. It was previously reported that elderly patients with ATAAD are preponderantly female and that a thrombosed FL is quite prevalent in this group [31, 32]. Rylski et al. analyzed 2,137 patients from the German Registry for ATAAD, and found that the risk of aortic dissection extending to the abdominal aorta decreased with age [33]. Similarly, we found that elderly patients were more likely to have a thrombosed FL and proximal dissection. This characteristic dissection morphology in elderly patients with ATAAD might have influenced the WBC count.

In addition to age and dissection extending to the iliac artery, multivariable analysis identified current smoking and absence of coronary ischemia as factors related to an elevated WBC count. Malenica at al. reported that healthy smokers (n = 56) had a significantly higher WBC count in comparison to healthy non-smokers (n = 100) (7.03 x10^3^/μL vs. 6.00 x 10^3^/μL, p<0.001) [34]. There have been no previous studies of the relation between coronary ischemia and WBC count in patients with acute aortic dissection, and we think the relatively small patient group included in our study might have influenced our result in terms of coronary ischemia.

Fan et al. reported an elevated WBC count (>11.0 x 10^3^/μL) to be an independent predictor of 30-day morality (hazard ratio, 3.31; p = 0.007) but that it was not related to late outcomes. We found no difference in in-hospital mortality or morbidity between the normal and elevated WBC count groups, and we found, unexpectedly, that late survival was worse in the normal WBC count group. There are several possible explanations for these discrepancies. First, patients with a WBC count >11.0 x 10^3^/μL accounted for 37.8% (216/570) of the Fan et al. study population [14] versus 54.7% of ours (because we excluded patients treated surgically more than 48 hours after symptom onset). Second, Fan et al. included patients who received medical therapy; 30% (171/570) did not undergo aortic repair [14]. Though the overall 30-day mortality was similar at 10.7% (61/579) in the Fan at al. study [14] and 7.5% (42/466) in our study, selection bias may have occurred in both studies, leading to the differences in outcomes. Cox proportional hazards analysis showed age to be the strongest factor related to post-discharge mid-term mortality (mean: 41.9 months), suggesting that a wide age difference between the 2 groups might have led to the difference in late survival in our study. We previously reported that FL patency is a predictor of outcomes in terms of survival and freedom from aortic events in ATAAD patients (n = 472; mean follow-up period: 81.3 months) [20]. The shorter follow-up period may explain why FL status was not identified as a factor related to late outcomes in our study reported herein.

Our study had several limitations. First, it was retrospective, and the sample size was relatively small, so a large-scale prospective study is needed to confirm our findings. Second, we did not assess the neutrophil/lymphocyte ratio, because the differential WBC count was measured in some, not all, patients. Third, we did not investigate prior medical therapy, including use of immunosuppressive agents and statins. Fourth, we did not measure blood cytokine or chemokine concentrations, and we did not assess infiltration of immune cells in aortic tissue samples. Further investigation is needed to elucidate the relation between local inflammation in the dissecting aorta and elevation of the peripheral blood WBC count.

## Conclusions

Our data suggest that both the patient age and dissection morphology (i.e., FL status and distally limited aortic dissection) influence the acute-phase systemic inflammatory response characterized by elevation of the WBC count in patients with ATAAD. Further experimental and clinical studies are needed to elucidate the mechanisms underlying the local and systemic inflammatory responses associated with ATAAD.

## Supporting information

S1 File Supplement. (XLS)
